# Coral reef fishes exhibit beneficial phenotypes inside marine protected areas

**DOI:** 10.1371/journal.pone.0193426

**Published:** 2018-02-22

**Authors:** Robert Y. Fidler, Jessica Carroll, Kristen W. Rynerson, Danielle F. Matthews, Ralph G. Turingan

**Affiliations:** 1 Department of Biological Sciences, Florida Institute of Technology, Melbourne, Florida, United States of America; 2 Fish and Wildlife Research Institute, Florida Fish and Wildlife Conservation Commission, St. Petersburg, Florida, United States of America; Department of Agriculture and Water Resources, AUSTRALIA

## Abstract

Human fishing effort is size-selective, preferentially removing the largest individuals from harvested stocks. Intensive, size-specific fishing mortality induces directional shifts in phenotypic frequencies towards the predominance of smaller and earlier-maturing individuals, which are among the primary causes of declining fish biomass. Fish that reproduce at smaller size and younger age produce fewer, smaller, and less viable larvae, severely reducing the reproductive capacity of harvested populations. Marine protected areas (MPAs) are extensively utilized in coral reefs for fisheries management, and are thought to mitigate the impacts of size-selective fishing mortality and supplement fished stocks through larval export. However, empirical evidence of disparities in fitness-relevant phenotypes between MPAs and adjacent fished reefs is necessary to validate this assertion. Here, we compare key life-history traits in three coral-reef fishes (*Acanthurus nigrofuscus*, *Ctenochaetus striatus*, and *Parupeneus multifasciatus*) between MPAs and fished reefs in the Philippines. Results of our analyses support previous hypotheses regarding the impacts of MPAs on phenotypic traits. Asymptotic length (L_inf_) and growth rates (*K*) differed between conspecifics in MPAs and fished reefs, with protected populations exhibiting phenotypes that are known to confer higher fecundity. Additionally, populations demonstrated increases in length at 50% maturity (L_50_) inside MPAs compared to adjacent areas, although age at 50% maturity (A_50_) did not appear to be impacted by MPA establishment. Shifts toward advantageous phenotypes were most common in the oldest and largest MPAs, but occurred in all of the MPAs examined. These results suggest that MPAs may provide protection against the impacts of size-selective harvest on life-history traits in coral-reef fishes.

## Introduction

In addition to driving population declines, increasing evidence suggests that fishing induces widespread alterations to life-history characteristics within harvested stocks. Rapid phenotypic shifts have been driven by the non-random nature of human fishing effort, which preferentially removes the oldest and largest individuals from any given population [1–2]. Intensive size-selective mortality generates directional selection pressure that favors individuals with smaller body-sizes [3–9] and those that reach reproductive maturity at smaller body-size and younger age [10–13]. Selective pressures from fishing can be strong enough to produce population-wide declines in asymptotic length, growth rate, and size- and age-at-maturation within contemporary timescales [4].

In many fishes, decreased female body-size in mature females correlates with reduced larval size-at-hatch, growth rate, feeding rate, and viability [14–15]. Additionally, females that reach sexual maturity at a young age breed for shorter periods and produce fewer, smaller eggs compared to conspecifics that mature later in life [16–17]. Declines in the abundance of old, large, highly-fecund individuals are therefore among the primary drivers of reductions in harvestable biomass in exploited stocks [18]. Decreased population size coupled with diminished reproductive capability can result in fishery collapse, best exemplified by the near disappearance of Atlantic cod (*Gadus morhua*) in southern Labrador and eastern Newfoundland, Canada, in the late 1980s; an event preceded by the widespread induction of reduced size- and age-at-maturation [7–8, 19–20]. Highlighting the long-term consequences of human-induced alterations to stock characteristics, *G*. *morhua* populations in the region have struggled to recover despite a fishing moratorium enacted in 1992 [21]. Only recently, after 25 years of fishing regulation and protection, have some *G*. *morhua* stocks displayed evidence of a significant rebound in population sizes [22], although they remain well below historical averages. Considering the rapid and devastating impacts of size-selective harvesting on the long-term viability of harvested populations, it is critical that life-history shifts be included in the design and assessment of programs aimed at managing fishery resources [23–24].

At present, a common component of coral-reef fishery management efforts are no-take marine reserves or marine protected areas (MPAs); spatially explicit zones in which fishing and other exploitative activities are prohibited. Between ~6,000–9,000 MPAs have been established worldwide, covering ~14.2 million km^2^ (3.9%) of the total ocean surface, with ~1.93 million km^2^ (0.53% of ocean surface) being fully no-take areas [25]. As no-fishing zones, MPAs eliminate size-selective fishing effort and the resulting directional selection pressure, theoretically arresting further proliferation of fishing-induced phenotypes within their borders [26–28]. Deleterious shifts in phenotypic frequencies may even be reversed by MPAs, as ecological forces generate selection gradients that favor larger body-size and greater age- and size-at-maturation [26–27, 29–30]. Controlled selection experiments have demonstrated that populations are able to restore historical body-size frequency distributions in 6–12 generations after the cessation of size-selective harvest [31]. It is unknown, however, if the processes observed in laboratory trials can occur in wild fisheries, or if they will be mitigated by natural predation and external influences, including habitat degradation and fishing pressure, outside of protected areas [7, 32].

The ability of MPAs to promote increased reproductive output per unit biomass is critical to their ability to meet their stated biological and economic objectives. Among the most important proposed benefits of MPAs is recruitment subsidy, the process by which excess larvae produced by healthy populations inside MPAs are transported to adjacent areas, increasing ecological resilience and improving fishery yields [33]. Consequently, MPAs must not only increase fish density, but also the relative fecundity of individuals within their borders, as relatively small populations inside MPAs must be able to supplement individuals lost to fishing in much larger spatial areas. Assessments of MPAs have demonstrated increased fish density, biomass, species diversity, and average body-size and age within MPAs compared to fished reefs [34–38]. Additionally, beneficial shifts in population structure towards the predominance of large-bodied individuals within MPAs have been observed in a multitude of reefs worldwide [35–36, 39]. However, it is unclear whether these observed trends are primarily the result of increased longevity in protected populations or are driven by shifts in phenotypic characteristics inside MPAs. Although studies have indicated that average batch fecundity may be increased inside MPAs compared to adjacent areas [40], egg production has also been shown to vary spatially due to a variety of influences unrelated to fishing pressure including water temperature and prey availability [41–42]. As a result, the ubiquity with which MPAs promote shifts in phenotypic frequencies within their borders remains in question.

In light of the growing anthropogenic pressure being placed on marine fisheries and the increasing use of MPAs as a management tool, a more thorough understanding of the effects of MPAs on fishery resources is essential. This study was designed to assess disparities in fitness-relevant phenotypes between protected and fished populations of three exploited reef-fish species in the Philippines. To do this, we use empirical data to compare the average asymptotic body size, growth rate, and length and age at 50% maturity of conspecific reef fish populations inside and outside of MPA borders.

## Materials and methods

**Fig 1:**
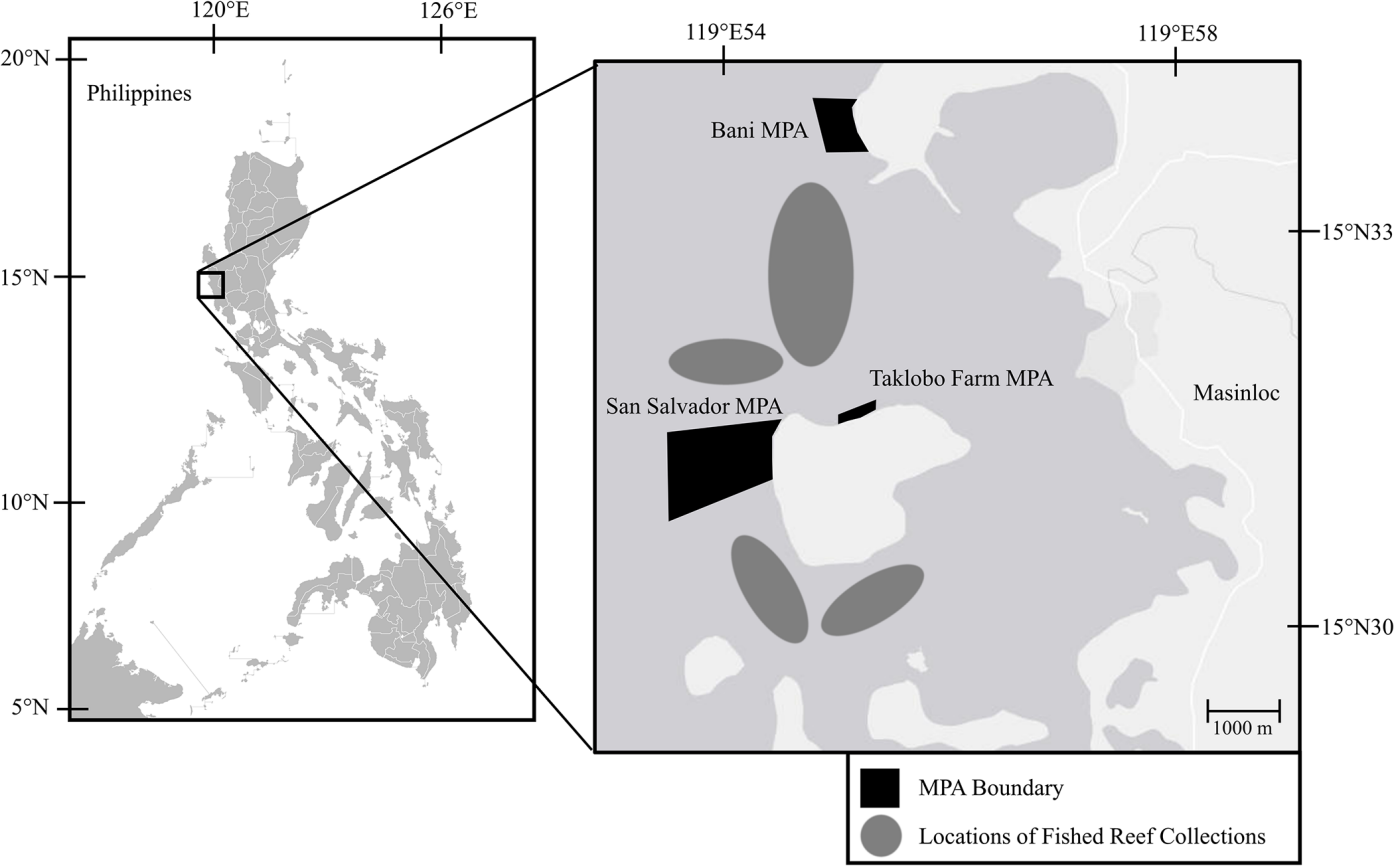
Sites of MPAs and fished reefs where collections of three coral-reef resident fishes occurred.

To test for disparities in fitness-relevant phenotypes of reef fishes between protected and harvested populations, individuals of three coral-resident fish species were collected in three MPAs and adjacent, fished reefs. Experimental fishes consisted of species directly targeted by fishers for food in the region: *Acanthurus nigrofuscus* (brown surgeonfish), *Ctenochaetus striatus* (lined bristletooth), and *Parupeneus multifasciatus* (manybar goatfish). Collections were conducted in Masinloc, Zambales Province, Luzon, Philippines (15° 31’ 40”N, 119° 57’ 38”E) between March and June of 2015. Collections were conducted in fished reefs and in three MPAs of varying size and age: Bani MPA (est. 2006; 50 ha), San Salvador MPA (est. 1989; 127 ha), Taklobo Farm MPA (est. 1989; 2 ha), (Fig 1). All MPAs are fully no-take zones and strongly enforced, with guards standing watch 18–24 hours a day with access to boats to confront any illegal fishing activity. Specimens of each species were collected throughout their observed body-size range at each site via scuba and spearfishing equipment. To ensure that individuals collected outside of MPAs were exposed to continuous fishing pressure, all collections in “fished reefs” took place at least 300m outside of MPA boundaries. Fish collection, handling, and otolith extraction and processing were conducted pursuant to the animal-handling protocol included in the Republic of the Philippines, Department of Agriculture Gratuitous Permit GP-0096-15 issued on June 01, 2015 in Quezon City, Philippines.

After collection, fish were sacrificed by immersion in ice while being transported to the laboratory for processing. Species-level identification was then confirmed, and the standard length (mm) of each fish was recorded. Gonads were removed, sexed, and staged using a protocol adapted from Murua et al. [43]. Gonads were classified into three stages: Stage 1 (immature); Stage 2 (sexually mature, but not fully engorged); or Stage 3 (mature and ripe, preparing to spawn). Both sagittal otoliths were removed, cleaned of adhering tissue, and suspended in an araldite epoxy mixture (82% Araldite 502, specific gravity 1.13, Electron Microscopy Sciences Inc.; 12% Hardener HY 956, Vantico Inc.), which was hardened at room temperature overnight. For consistency, the left otolith was used for analysis whenever possible. After hardening, three 0.45–0.70mm sections were cut from the center of each otolith using a Buehler Isomet low-speed saw. Following sectioning, each otolith was secured to a slide using a toluene-based liquid mounting medium, and subsequently aged on a Leica MZ9.5 stereomicroscope under transmitted light. Ageing was conducted by enumerating opaque rings (counted as annuli) along the ventral edge of the sulcus acousticus. Alternating patterns of translucent and opaque rings have been validated as annuli in *A*. *nigrofuscus* [44] and *C*. *striatus* [45]. Due to their structure, ageing analyses were conducted on whole otoliths rather than sections for *P*. *multifasciatus*. Otoliths were submerged in water within a black petri dish, and analyzed using a Leica MZ9.5 stereomicroscope under reflected light. Annual deposition is unvalidated for *P*. *multifasciatus*; however, the seasonality of opaque zone formation was consistent with annual deposition.

To test for disparities in asymptotic lengths and growth rates between populations, Von Bertalanffy growth models were developed for populations collected within each MPA and associated fished reefs. The average length (*l*) of fish at time (*t*) was calculated as:

$$l_{t}=L_{inf}(1-e^{-k(t-t_{0})})$$

where *t*_*0*_ is the theoretical time at which length = 0, L_inf_ is the mean maximum length or “asymptotic length” where growth = 0, and *K* is the rate (years^-1^) at which L_inf_ is attained. Von Bertalanffy growth models were compared using the likelihood-ratio method [46]. This analysis tests for differences in the average asymptotic length (L_inf_), growth rate (*K*), and time at length 0 (*t*_*0*_) between populations. Due to variations in the availability of specimens less than a year old and limited sample sizes within MPAs, Von Bertalanffy growth models occasionally produced unrealistic growth patterns at early ages within individual sites. Consequently, an additional individual of age “0” and size “0” was added to each population in order to properly set growth functions. Statistical analyses were conducted using the *fishmethods* package in R statistical software [47].

To estimate the length (L_50_) and age (A_50_) at which 50% of individuals were mature within each population, logistic regression models were fit to data regarding the percentage of individuals found to be mature within age classes (year) and size classes (every 2mm). In addition, 95% confidence intervals around these estimates were created by bootstrapping with 999 iterations. Analyses were conducted using the *glm* function and *FSA* package in R statistical software [48].

## Results

A total of 569 specimens were collected from MPAs and associated fished reefs (S1 Table). Populations of *P*. *multifasciatus* were not robust enough in the Taklobo Farm MPA to conduct collections, and therefore were only collected from the Bani and San Salvador MPAs. In the following descriptions of statistics, the term “MPA” will be used when referring to protected populations, whereas “FR” will be used when referring to fished reefs.

Von Bertalanffy growth models revealed considerable variation in life-history traits between species and sites. Populations of *A*. *nigrofuscus*, *C*. *striatus*, and *P*. *multifasciatus* all exhibited disparities in growth parameters between at least one MPA compared to fished reefs. Significant differences were concentrated in the two oldest MPAs in the region: San Salvador and Taklobo Farm (both 26 years). Differential life-history traits were, however, occasionally observed between populations inside and outside of the Bani MPA. Statistical results of comparisons of asymptotic lengths (L_inf_), growth rates (*K*), and time at length 0 (*t*_*0*_) for all MPA-fished reef pairs are listed in Table 1.

**Table 1:** Results of statistical comparisons of asymptotic length (L_inf_; SL, mm), growth rates (*K*), and time-at-age-0 (*t*_*0*_) between populations inside MPAs (MPA) and associated fished reefs (FR), using the likelihood ratio method of Kimura [46]. Length values have been rounded to the nearest mm. Values for each variable in MPAs and fished reefs are provided, along with χ2 statistics and associated p-values. Significant results are indicated by bold font.

		Bani				San Salvador				Taklobo Farm			
Species	Trait	MPA	FR	X2	P	MPA	FR	X2	P	MPA	FR	X2	P
*Acanthurus*	Lin_f_	80	78	0.48	0.488	**89**	**78**	**11.52**	**0.001**	**84**	**78**	**5.60**	**0.018**
*nigrofuscus*	K	1.17	0.89	1.98	0.159	0.82	0.89	0.13	0.718	0.98	0.89	0.21	0.647
	t_0_	-0.05	-0.28	3.36	0.067	-0.18	-0.28	0.35	0.554	-0.11	-0.28	1.32	0.251
*Ctenochaetus*	Lin_f_	**132**	**119**	**6.00**	**0.014**	125	119	2.56	0.110	**126**	**119**	**4.82**	**0.028**
*striatus*	K	0.66	0.72	0.05	0.823	**0.49**	**0.72**	**3.97**	**0.046**	0.69	0.72	0.06	0.806
	t_0_	-0.16	-0.12	0.68	0.410	-0.36	-0.12	1.68	0.195	-0.05	-0.12	0.28	0.597
*Parupeneus*	Lin_f_	157	135	2.76	0.097	**173**	**135**	**5.29**	**0.021**	N/A	135	N/A	N/A
*multifasciatus*	K	0.22	0.29	0.68	0.410	**0.14**	**0.29**	**4.83**	**0.028**	N/A	0.29	N/A	N/A
* *	t_0_	-0.42	-0.30	0.06	0.806	-1.09	-0.30	1.56	0.212	N/A	-0.30	N/A	N/A

**Fig 2:**
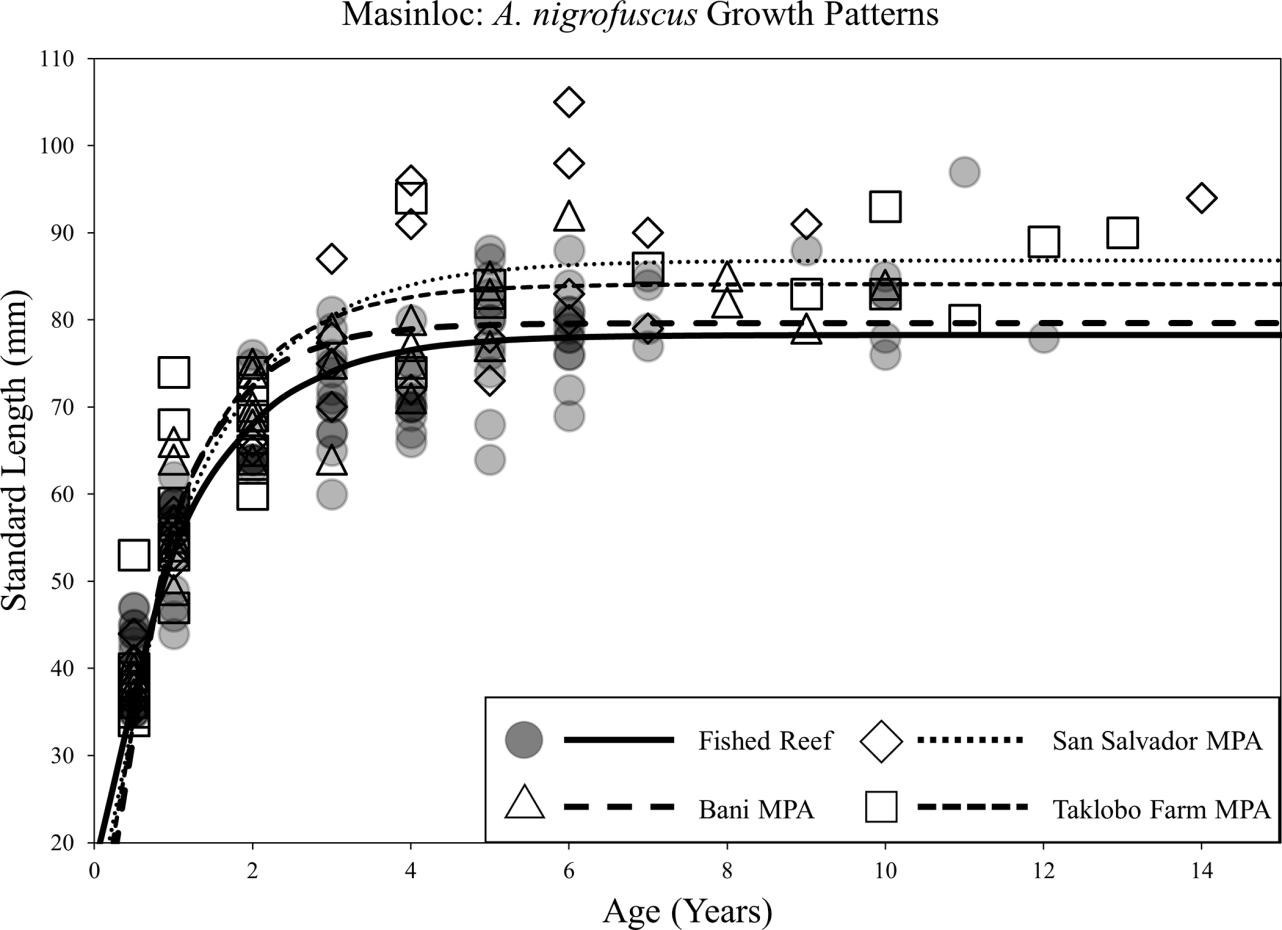
Scatterplot of the length-at-age of *A*. *nigrofuscus* individuals caught inside and outside of the Bani, San Salvador, and Taklobo Farm MPAs in Masinloc, and associated Von Bertalanffy growth curves for protected and fished populations.

**Fig 3:**
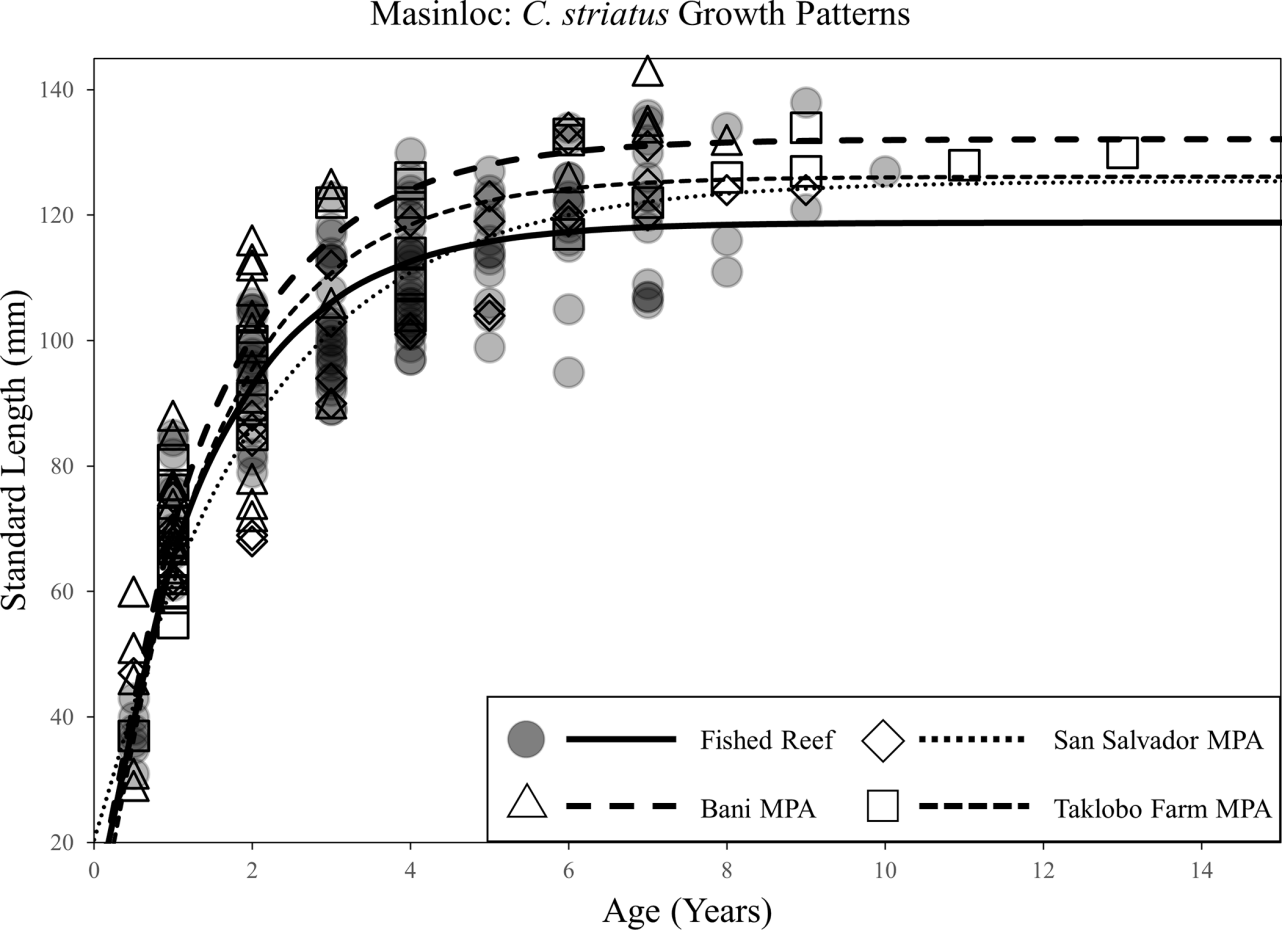
Scatterplot of the length-at-age of *C*. *striatus* individuals caught inside and outside of the Bani, San Salvador, and Taklobo Farm MPAs in Masinloc, and associated Von Bertalanffy growth curves for both protected and fished populations.

**Fig 4:**
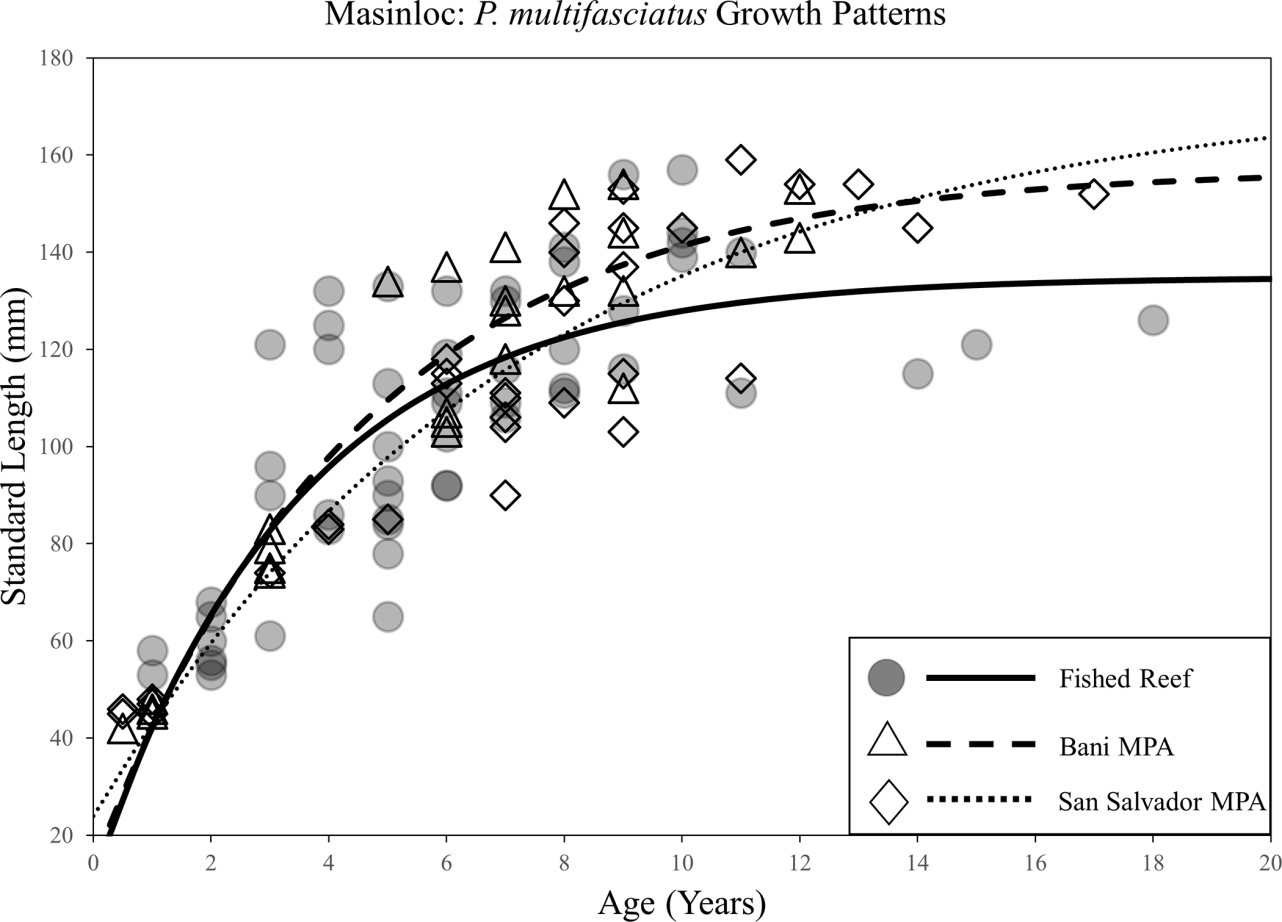
Scatterplot of the length-at-age of *P*. *multifasciatus* individuals caught inside and outside of the Bani and San Salvador MPAs in Masinloc, and associated Von Bertalanffy growth curves for both protected and fished populations.

Significant increases in L_inf_ were observed in populations of *A*. *nigrofuscus* inside MPAs compared to fished reefs for both the San Salvador MPA and the Taklobo Farm MPA (Fig 2). Asymptotic lengths inside these MPAs were 11mm (14%) and 6mm (8%) greater than populations in fished reefs, respectively. Populations of *C*. *striatus* exhibited higher L_inf_ values in the Bani and Taklobo Farm MPAs, with asymptotic lengths being 13mm (11%) and 7mm (6%) greater than those in fished reefs (Fig 3). Populations of *P*. *multifasciatus* demonstrated significantly higher asymptotic length inside the San Salvador MPA, with protected stocks reaching body-sizes 38mm (28%) greater than those in fished reefs (Fig 4). Shifts in the growth rate (*K*) of conspecifics between MPAs and fished reefs occurred only for *C*. *striatus* and *P*. *multifasciatus*, with both species exhibiting lower *K* values inside the San Salvador MPA compared to fished reefs (Figs 3 and 4). Slower growth rates were rarely associated with significant differences in asymptotic length, with shifts in both life-history traits occurring simultaneously only once (*P*. *multifasciatus*, San Salvador MPA).

Observed values of age- and length-at-maturation are illustrated in S1–S3 Figs, and the results of logistic regression models for length (L_50_) and age (A_50_) at 50% maturity can be found in Tables 2 and 3, respectively. In general, the 95% confidence intervals around estimates of L_50_ and A_50_ for populations in MPAs and associated fished reefs were overlapping. However, the confidence intervals for L_50_ did not overlap for *P*. *multifasciatus* at either of the two MPAs from which it was collected and fished reefs, and for *A*. *nigrofuscus* in the Taklobo Farm MPA compared to fished reefs, with L_50_ values being greater inside MPAs in all cases (Table 2). Conversely, the confidence intervals for A_50_ overlapped in all but one instance (*C*. *striatus*, Bani MPA), which demonstrated A_50_ values lower inside the MPA compared to those in fished reefs (Table 3).

**Table 2:** Estimated length at 50% maturity (L_50_) and associated 95% confidence intervals (CI) for each population. Values have been rounded to the nearest mm. Instances where 95% CIs did not overlap between any MPA and associated fished reef are indicated by bold font.

	*A*. *nigrofuscus*			*C*. *striatus*			*P*. *multifasciatus*		
MPA/ Fished Reef	L_50_	CI Lower	CI Upper	L_50_	CI Lower	CI Upper	L_50_	CI Lower	CI Upper
Bani	57	49	61	84	75	88	**105**	**92**	**111**
San Salvador	58	57	62	70	67	83	**102**	**94**	**110**
Taklobo Farm	**64**	**62**	**66**	79	76	88	N/A	N/A	N/A
Fished Reef	59	57	61	84	76	89	85	73	89

**Table 3:** Estimated age at 50% maturity (A_50_) and associated 95% confidence intervals (CI) for each population. Instances where 95% CIs did not overlap between any MPA and associated fished reef are indicated by bold font.

	*A*. *nigrofuscus*			*C*. *striatus*			*P*. *multifasciatus*		
MPA/ Fished Reef	A_50_	CI Lower	CI Upper	A_50_	CI Lower	CI Upper	A_50_	CI Lower	CI Upper
Bani	1.02	0.53	1.49	**0.98**	**0.91**	**1.03**	5.02	4.00	6.48
San Salvador	1.09	1.01	1.53	1.01	0.94	1.49	5.71	4.53	6.63
Taklobo Farm	1.54	1.02	1.97	1.11	1.05	1.53	N/A	N/A	N/A
Fished Reef	1.06	0.99	1.50	1.49	1.15	1.91	4.11	3.37	5.14

## Discussion

A direct comparison of life-history characteristics in three reef fishes revealed considerable variation in fitness-relevant phenotypes of conspecifics, with disparities in asymptotic length (L_inf_) and/or growth rates (*K*) being observed in each species within at least one MPA when compared to fished reefs. This result suggests that MPAs can promote shifts in the frequency of large-bodied fish within their borders with relative consistency across a multitude of species. The impact of MPA establishment on maturation schedules in protected populations, however, was much less consistent. Although estimates of length at 50% maturity (L_50_) were larger inside two MPAs for *P*. *multifasciatus* and one MPA for *A*. *nigrofuscus*, L_50_ values were not significantly different for *C*. *striatus* in any MPA. In addition, age at 50% maturity (A_50_) differed only between the Bani MPA and fished reefs for *C*. *striatus*, but was lower inside the MPA than in fished reefs. That fish inside MPAs did not delay maturation until later ages may mitigate some of the potential reproductive benefits of larger asymptotic length and larger size at maturation. However, the increase of large-bodied, larger-maturing individuals is still likely to result in an overall increase in relative reproductive potential inside MPAs compared to adjacent, fished reefs.

The age of MPAs appeared to play a substantial role in whether phenotypes differed between protected and harvested populations. Significantly larger L_inf_ values were observed predominantly in the two oldest MPAs (San Salvador [26 years; *A*. *nigrofuscus*, *P*. *multifasciatus*], and Taklobo Farm [26 years; *A*. *nigrofuscus*, *C*. *striatus*]), although *C*. *striatus* also exhibited higher asymptotic length inside the Bani MPA (9 years). These results support theoretical predictions regarding the temporal scale required for populations inside MPAs to demonstrate phenotypic recovery, with demonstrable changes in life-history frequencies requiring many years to appear [31, 49–50]. The size of MPAs, however, did not seem to be a contributing factor to phenotypic variation between sites. Populations in which life-history traits differed from fished reefs were found in both the largest (San Salvador [127 ha]) and smallest (Taklobo Farm [2 ha]) MPAs in the study, suggesting that long-term protection can promote beneficial changes across a range of MPA sizes. This result is unexpected, as movement into fishing grounds increases the selective force of fishing on protected populations, thereby dampening the benefits of protected areas [28, 51]. Species with small territories such as *C*. *striatus* (12m^2^ [52]), are not likely to traverse MPA boundaries even in the smallest MPAs examined here. In contrast, individuals of *A*. *nigrofuscus* are known to conduct daily feeding and spawning migrations from sheltered sites that can range from 300m [53] to 1.5km [54], and *P*. *multifasciatus* roams areas up to 245m^2^ daily [55], making it unlikely that populations of these species are distinct between MPAs and fished reefs. It is also possible that the age and size of MPAs play synergistic roles in promoting phenotypic shifts in exploited populations. For example, increases in L_inf_ for *A*. *nigrofuscus* were greater in the San Salvador (127 ha; 26 years) MPA than the Taklobo Farm MPA (2 ha; 26 years), and asymptotic length of *C*. *striatus* was higher in the Bani MPA (50 ha; 9 years) than in the Taklobo Farm MPA. The effects of MPA size and age on the life-history variations will likely vary based on the genetic, plastic, and density-dependent processes controlling phenotypic shifts occurring inside MPAs [6].

There is considerable debate regarding the mechanism underlying phenotypic changes observed in exploited fish stocks. Specifically, the relative contributions of environmentally- or density-dependent phenotypic plasticity and fixed genetic alterations remain in question [56]. Although fish body-size and maturation are plastic in many species [31], the intensive harvest rates and size-selectivity of fisheries [5] and demonstrated heritability of life-history traits in fishes [3] provide the necessary conditions to produce genetic shifts in growth and maturation patterns [57]. Additionally, alterations to life-history traits have been shown to correlate with concurrent shifts in allelic frequencies [52], suggesting that phenotypic shifts in wild fish stocks have some genotypic basis. The distinction between phenotypic plasticity and genetics is critical to the recovery potential of exploited populations. Genetic changes are expected to be slower and more challenging to reverse than plastic responses [58–60], affect fundamental population dynamics [61], and impact resilience to environmental stress [62]. However, genetic shifts will be necessary for beneficial traits to extend to fished reefs through spillover and recruitment subsidy [26, 28]. Consequently, genotypic responses within MPAs would represent long-term benefits throughout interconnected populations, while plastic changes would only manifest inside MPAs, and may be rendered null if protection is ceased.

Given that the populations examined here are interconnected through both pelagic larval stages and adult movement patterns, genetic stratification between MPAs and fished reefs is unlikely. This suggests that observed phenotypic changes inside MPAs are more likely the result of plastic responses to the removal of fishing mortality. Conversely, the prevalence of life-history shifts in older MPAs indicates that multiple generations are required for alterations to growth rates and body sizes to occur, supporting a genotypic basis of growth and maturation phenotypes. It is also possible that extended periods of protection may be necessary for populations to reach densities great enough to induce plastic responses, in which case density-dependent growth may be primarily responsible for changes in life-history characteristics. Density-dependent mechanisms would help to explain life-history shifts in the Taklobo Farm (2 ha) and Bani (50 ha) MPAs, but not in the larger San Salvador MPA (127 ha). It is also conceivable that variations in environmental parameters such as habitat or food availability between MPAs and fished areas may be responsible for observed shifts in growth patterns [44]. However, no distinct differences in coral cover or algal prevalence were observed between sites during the course of the study. Alternatively, increased growth and egg production inside MPAs may have been driven by differential stress levels between fished and protected populations, as fish flight initiation distance is positively associated with fishing pressure and decreases significantly inside MPAs [63–64]. Populations undergoing constant fishing pressure therefore spend more time avoiding fishing mortality and less time actively foraging, limiting the amount of potential energy that can be allocated to growth and reproduction. This interpretation is consistent with studies that have suggested that fishing pressure actively selects against bold behavioral types, which are often associated with increased reproductive output [65].

Although it appears that *A*. *nigrofuscus*, *C*. *striatus*, and *P*. *multifasciatus* have exhibited positive responses to MPA establishment, species-specific analyses across a wide range of taxa will be critical in determining the overall effect of MPAs on reef-fish communities, as there are many factors that may modulate the phenotypic impacts of MPAs. First, family-level susceptibility to fishing pressure [66], as well as recovery rates of individual fish families [67], differ significantly. Second, harvest intensity varies greatly depending on the species in question and the locality of the study [66, 68]. Differential harvest rates will affect the degree to which fishing-induced phenotypic shifts occur within a population, which will not only determine current population sizes and reproductive potential, but also the rate at which fisheries are likely to recover after the cessation of harvest pressure [15, 69]. Third, responses to protection depend on species-specific movement and behavioral patterns, economic value, body size, habitat depth range, and schooling behavior [51, 70]. Fourth, rates of evolutionary change have been shown to vary across conspecific fishery stocks that exhibit different somatic growth rates [71]. As a result, the rates and magnitude of life-history shifts are likely to differ not only across taxa, but also between disparate populations of any given species. Finally, traditional MPA theory predicts greater biological benefits from older and larger MPAs, necessitating the inclusion of the size and age of MPAs in assessments of their impact on fish life-history traits. Future empirical investigations and modelling studies will be critical in advancing our understanding of how populations will respond to spatially explicit cessation of harvest mortality, and how MPAs might best be employed to maximize their benefit to fishery stocks.

The restoration of healthy fishery populations is not only important for resource management, but is also essential for future ecological sustainability in Philippine coral reefs. Fishing pressure is considered a driving force behind deleterious ecological shifts within marine ecosystems [72], as the preferential removal of commercially targeted species significantly reduces functional diversity in exploited reefs [73]. Size- and age-truncated fishery stocks recover more slowly after fishing moratoria, as high reproductive investment at small size and early age diminish relative population growth under natural mortality scenarios [58], increasing the vulnerability of fisheries to environmental stress. MPAs have demonstrated the ability to act as buffers against mass mortality from isolated events [74], but their ability to combat population collapses against repeated, unpredictable environmental perturbations is less understood. As global climate patterns become increasingly variable, it is essential that MPAs promote the development of robust, highly fecund populations that can better withstand environmental heterogeneity [75]. Here, we have provided evidence of disparities in phenotypic frequencies between conspecific fish populations inside and outside of MPAs. Continued studies are necessary to improve our understanding of how ubiquitous these trends are across various MPAs, and whether phenotypic shifts will be substantial enough to promote long-term population resilience in harvested coral-reef fishes in the face of mounting anthropogenic pressures.

## Supporting information

S1 Dataset Dataset used for all analyses. In the tables, “F” represents females, “M” males, and “U” unknown gender. For gonad stages, “1” represents immature, “2” mature, and “3” mature, engorged and preparing to spawn. The term “STG” refers to individuals that had gonads damaged during collection, making sexing and staging unreliable.
(XLSX)

S1 Table Total counts of all individuals collected from MPAs and fished reefs. Fish are divided by gender (male [M] or female [F]) and by site. Individuals listed as unknown (U) either had their gonads significantly damaged during collection, or were juveniles, making gender identification unreliable. The term “T” denotes the total number of fish caught in any given category.
(XLSX)

S1 Fig Relative maturity by age (years) and size (standard length, mm) of collections of *A*. *nigrofuscus*. (TIF)

S2 Fig Relative maturity by age (years) and size (standard length, mm) of collections of *C*. *striatus*. (TIF)

S3 Fig Relative maturity by age (years) and size (standard length, mm) of collections of P. multifasciatus. (TIF)
