# Supplementary figures and images for: Coral reef fishes exhibit beneficial phenotypes inside marine protected areas
Source: PLoS One. 2018 Feb 22;13(2):e0193426. doi: 10.1371/journal.pone.0193426 (PMC5823445; doi:10.1371/journal.pone.0193426)

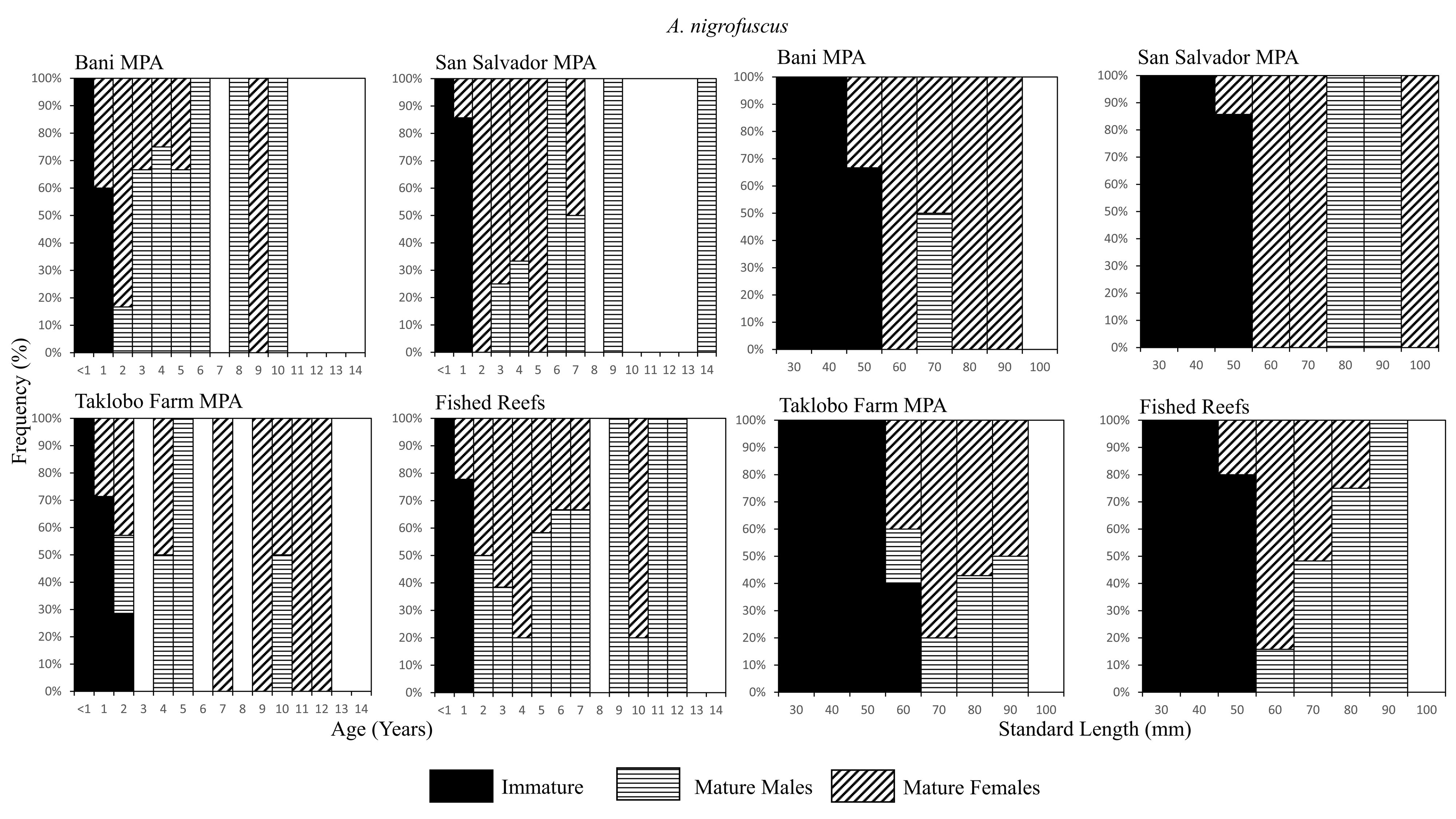

Supplement: S1 Fig — (TIF) [file pone.0193426.s003.TIF]

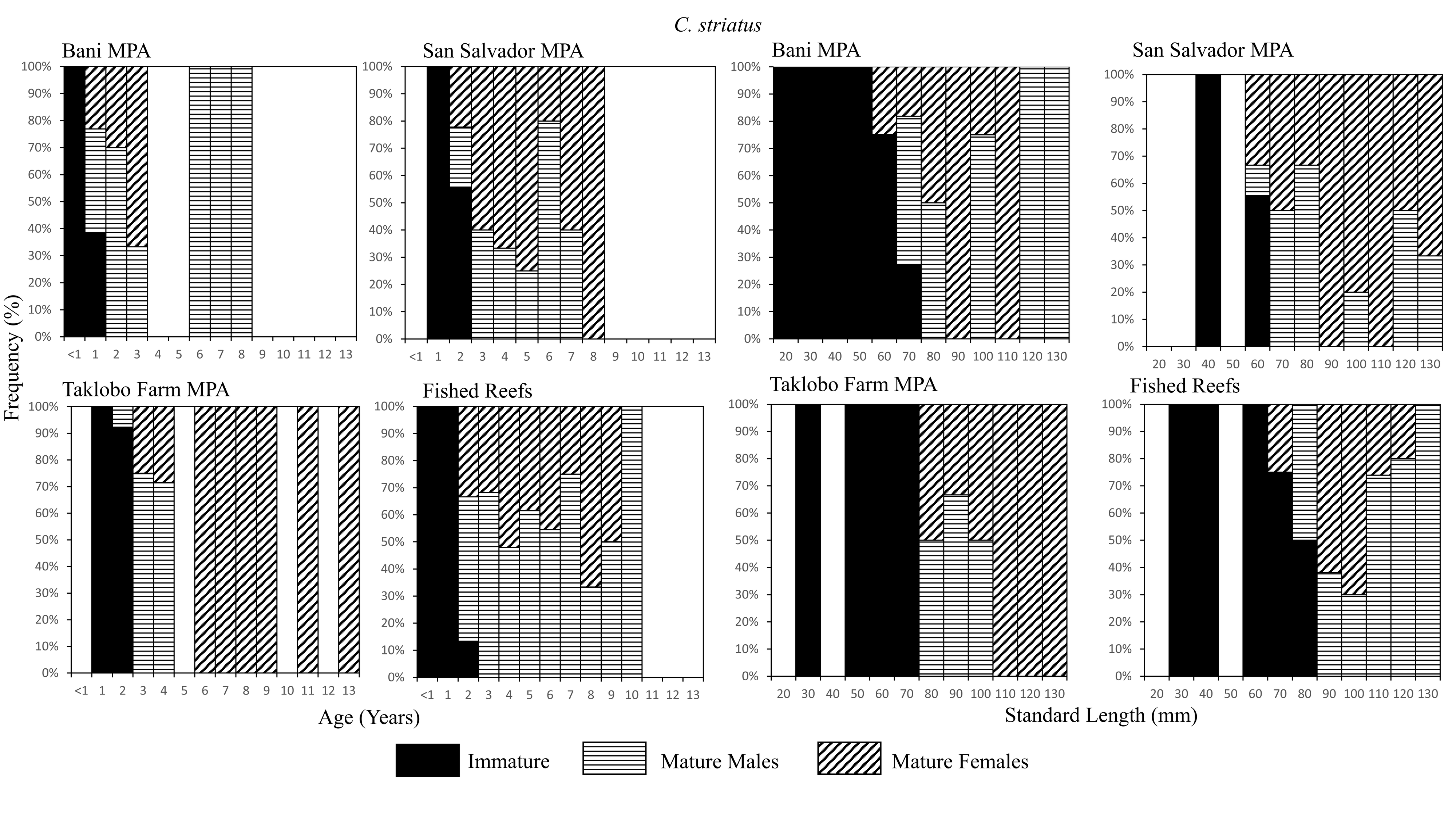

Supplement: S2 Fig — (TIF) [file pone.0193426.s004.TIF]

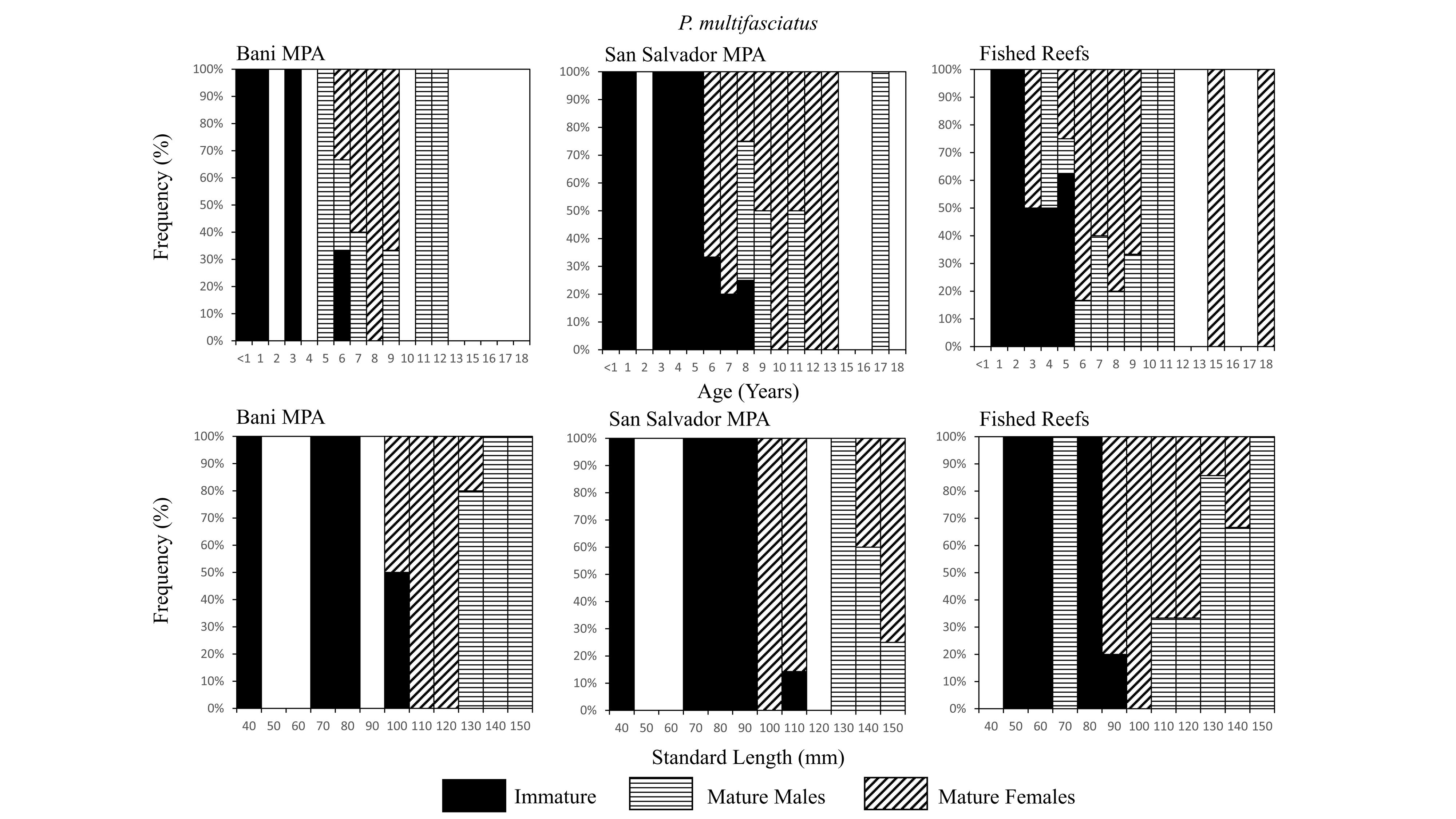

Supplement: S3 Fig — (TIF) [file pone.0193426.s005.TIF]
